# Exploring astrobiology using *in silico* molecular structure generation

**DOI:** 10.1098/rsta.2016.0344

**Published:** 2017-11-13

**Authors:** Markus Meringer, H. James Cleaves

**Affiliations:** 1 Earth Observation Center (EOC), German Aerospace Center (DLR), Münchner Straße 20, 82234 Oberpfaffenhofen-Wessling, Germany; 2 Earth-Life Science Institute, Tokyo Institute of Technology, 2-12-IE-1 Ookayama, Meguro-ku, Tokyo 152-8551, Japan; 3 Institute for Advanced Study, Princeton, NJ 08540, USA; 4 Blue Marble Space Institute of Science, 1515 Gallatin Street NW, Washington, DC 20011, USA; 5 Center for Chemical Evolution, Georgia Institute of Technology, Atlanta, GA 30332, USA

**Keywords:** chemical space, prebiotic chemistry, origin of life, biochemical evolution, molecular graph, chemoinformatics

## Abstract

The origin of life is typically understood as a transition from inanimate or disorganized matter to self-organized, ‘animate’ matter. This transition probably took place largely in the context of organic compounds, and most approaches, to date, have focused on using the organic chemical composition of modern organisms as the main guide for understanding this process. However, it has gradually come to be appreciated that biochemistry, as we know it, occupies a minute volume of the possible organic ‘chemical space’. As the majority of abiotic syntheses appear to make a large set of compounds not found in biochemistry, as well as an incomplete subset of those that are, it is possible that life began with a significantly different set of components. Chemical graph-based structure generation methods allow for exhaustive *in silico* enumeration of different compound types and different types of ‘chemical spaces’ beyond those used by biochemistry, which can be explored to help understand the types of compounds biology uses, as well as to understand the nature of abiotic synthesis, and potentially design novel types of living systems.

This article is part of the themed issue ‘Reconceptualizing the origins of life’.

## 1. Introduction

To a first-order consideration, organic structure space is composed of all molecules containing carbon which satisfy Lewis electron pairing rules [1]. This is practically limited to exclude molecules which contain some types of structural features [2], for example those which would render compounds unstable in water. Most considerations of this space include some practical restrictions, such as having the output molecules being ‘meaningful’ in terms of pharmacological space [3].

Organic structure space is estimated to be extremely large [4], of the order of 10^33^ to 10^180^ unique structures [3,5]. Organic formula space belies the complexity of organic structure space: a single molecular formula can represent many structural isomers [6,7], although the number per unique formula may be highly variable (see for example [8,9]). Today, this plentitude of chemical structures offers scientists in medicinal chemistry, pharmaceutical research and biotechnology an almost endless array of possibilities to design new drugs and materials. Life itself has likely, during all steps of its evolution, optimized its biochemical processes within the vast organic structure space available to it, and a better understanding of life's biomolecular foundations in relation to the surrounding chemical space might be one key to understanding its origin.

From its very beginnings, the development of algorithms for the enumeration of chemical space was closely related to NASA's exobiology activities. The complete and non-redundant generation of all connectivity isomers, corresponding to a given molecular formula, was part of the DENDRAL (a portmanteau of ‘dendritic algorithm’) program, established in the mid-1960s [10,11]. In the 1970s and 1980s, mathematicians provided new techniques to increase the efficiency of the first approaches [12,13], and, starting in the 1990s, implementations became available as software packages for personal computers [14]. Recently, these methods have been rediscovered for applications in astrobiology and origins of life research, particularly for generating and analysing virtual chemical compound libraries of amino acids and nucleotide analogues [8,9,15], although the scope of their potential application is much wider.

There are numerous settings across the Universe in which abiotic organic synthesis occurs [16–19]. While catalogues of the organics in some of these settings, such as the interstellar medium, are of relatively small size (of the order of 190 unique structures from mass 13 to 840 amu (see, for example, http://www.astro.uni-koeln.de/cdms/molecules/), others are remarkably complex. For example, recent studies of carbonaceous chondrites suggest that the number of unique identifiable molecular formula organic compounds (over the mass range from 150 to 1000 amu) may be of the order of 14 000–50 000 [20], and that these could correspond to several million unique chemical structures. Studies using similar techniques examining the products of Miller–Urey electric discharge experiments [21], Titan tholins and hydrogen cyanide (HCN) polymers [22,23] have found similarly large numbers of unique formulae. Unfortunately, while one-dimensional high-resolution mass spectrometry is able to determine exact masses and therefore molecular formulae with a great deal of accuracy, as mentioned above there is an enormous amount of structural isomerism in organic chemistry, thus each detected unique formula may be representative of an as-yet-unknown number of structural isomers. It should be noted that the mass fragmentation spectrum is an additional powerful tool which can be used to potentially distinguish structural isomers.

In common with the organics found in abiotic simulations and some carbonaceous meteorites, the number of relatively low-molecular-weight compounds which biology, as a whole, is capable of producing is very large; for example, the *Dictionary of Natural Products* listed approximately 214 000 compounds as of March 2009 [24]. By contrast, the common core of modern biochemical metabolism is remarkably small, of the order of 500 to a few thousand compounds [25,26].

These observations have some bearing on the origin and evolution of life. First, as it is now clear that the abiotic organic chemical space of the Universe is very large, it must be acknowledged that far less is known about prebiotic chemistry, the chemistry which led to the origin of life, than has perhaps been presumed [27]. Second, as many of the secondary metabolites which make up the grand diversity of biological natural products come from metabolic transformations mediated by evolutionarily derived enzymes, and the chemical space to be explored is so incredibly large, it is likely that there is little overlap of these compounds with abiological samples, and possible that an independent biochemistry might develop along a very different chemical trajectory.

*In silico* chemical structure libraries have already been generated to examine a variety of origins and astrobiology-related research questions. We review here some approaches and general methods, and some results and ongoing work from our research group.

## 2. Material and methods

It was recognized some time ago that organic chemical structures are essentially graphs [28–30], thus the key mathematical concept to represent structural formulae of organic compounds is provided by graph theory. In a molecular graph, nodes correspond to atoms of a chemical structure and edges encode covalent bonds between atoms [31,32]. For this purpose, the nodes are labelled by the chemical identity of the represented atoms along with additional higher order information describing, for example, the state of the atom, e.g. its charge, whether it has associated paired or unpaired electrons, etc., while edges can also represent the multiplicity of bonds, i.e. whether they are single, double or triple bonds. More refined models are able to handle mesomerism and assign aromatic bonds [33].

Indeed, many concepts from graph theory are well suited to describe the structural properties of chemical compounds [34,35], which can also be related to chemical properties. For instance, the principle of subgraph relationships can be used to identify substructures or functional groups of a molecule. Other measures, such as topological distances between nodes or lengths of cycles, can be used to calculate invariants of molecular graphs, so-called topological indices or more general molecular descriptors that are well suited to serve in combination with machine learning techniques for predicting physico-chemical or biological properties via quantitative structure–property or structure–activity relationships (QSPR, QSAR) [36,37].

The increasing demand to generate, store, screen and process large sets of organic compounds using computers and to predict their properties with high accuracy has established new scientific disciplines such as mathematical chemistry and chemoinformatics [33,38]. We use methods honed in these disciplines to approach questions posed by astrobiology and the search for the origins of life. In particular, we are studying the entirety of monomers that are capable of building life's functional biopolymers, proteins and RNA/DNA [8,9,15], as well as the larger chemical spaces these special compound classes are a subset of.

Such studies require at least two steps: first, defining the chemical space to be investigated and generating the molecular libraries of its constituents for further computational processing, and, second, deriving and testing models that simulate how life on Earth or elsewhere in the Universe could choose or has chosen its monomeric building blocks during chemical and early biological evolution.

An obvious and simple way to retrieve molecular libraries of interest would be to query chemical structure databases, such as PubChem (https://pubchem.ncbi.nlm.nih.gov), Beilstein (now distributed as Reaxys, http://www.reaxys.com) or the Chemical Abstract Service (CAS) registry (https://www.cas.org, which presently contains more than 127 million unique organic and inorganic compounds). Although these databases contain a large repository of to-date synthesized compounds and analysed natural products, they will always suffer a certain bias of human interest, e.g. pharmaceutical utility or other economic ambitions, and of course the extraordinary size of organic chemical space (see above).

An alternative, somewhat more technical, though much more comprehensive, way is to generate the molecules of interest by means of dedicated algorithms and computer programs, so-called structure generators. Although formerly being the domain of highly specialized chemoinformaticians, computational chemists and chemical mathematicians, there are now databases available which provide the content produced by structure generators (see, for example, [2,3,39,40]). But, again, the main purpose of these projects was to explore and provide promising structures for virtual drug screening rather than providing dense coverage of the chemical space relevant to life's origins, or even dense coverage of chemical space as an interesting phenomenon *per se*.

Nevertheless, chemical databases are an important means to verify the completeness and significance of custom-generated specialty libraries [8,9]. On the one hand, there should be no structures retrieved from databases that are not included in custom-generated libraries, and, on the other hand, the overlap of custom-generated libraries with extant databases should ideally be small, justifying the computationally more demanding approach. Owing to the vastness of organic chemical space, so far these two considerations have proved valid in our experience.

There are two principally different ways to generate molecular graphs. One natural way is to begin with a set of starting compounds and iteratively apply a set of graphically coded chemical reactions to the starting materials and the upcoming intermediates. This process has either natural or artificial stopping criteria, e.g. if no more new reaction products are generated, or a certain number of iterations is reached, structure generation terminates. Reaction-based structure generation has been implemented using reaction schemes [41], which describe generic chemical reactions by a reaction centre graph and changes occurring during the reaction, closely related to the Dugundji–Ugi model [42] and the approach of Temkin *et al.* [43]. Graph grammars, as introduced in [44], have already been applied to problems in origins of life research [45–47].

However, reaction-based structure generation is determined by the seed compounds and the reactions applied. This approach can be especially effective if the types of reactions and reactants allowed within a system are well understood or constrained. However, if even a few starting compounds or side-reactions are overlooked, this approach risks missing significant numbers of potential reaction products. For some systems, especially those in which reactions or reactants are poorly constrained, for example in exploring the development of systems in which catalysis could facilitate unusual transformations, or for systems which could be seeded by unusual compounds derived from highly energetic processes, such assumptions are better avoided in order to obtain an output that is especially permissive of possibilities.

An alternative method addressing these concerns is a formula-based structure generation starting from a molecular formula and optional (but generally useful) structural constraints. In this method, the corresponding connectivity isomers are generated completely and non-redundantly. The algorithmic principle applied here is called *orderly generation* [12,48].

For our studies, we have used a well-vetted and -used structure generator, MOLGEN 5 [49], which provides a wide variety of structural constraints and further features that facilitate the construction of *in silico* compound libraries. This program permits the use of so-called fuzzy formulae that allow ranges to be specified for the numbers of atoms for each chemical element. This makes it possible to explore multiple molecular formulae in one program call. Another useful and time-saving feature of MOLGEN 5 is the ability to include lists of forbidden substructures (badlists) that can be passed as input to the program. Using such badlists, it is possible to suppress unlikely, typically unstable chemical structures in the output datasets. Comparison of the output datasets with published databases again offers an opportunity to vet whether the badlist substructures represent valid restrictions on real-world chemistry. This process of generation and comparison can be repeated iteratively. If valid structures are found in databases containing compounds that have, in fact, been synthesized and/or isolated from the chemical literature, then the badlist is too restrictive.

It should be noted that formula-based structure generation does not formally take into account synthesizability, although this criterion can be evaluated in the output structure sets using various programs, for example the commercially available SYLVIA (estimation of the synthetic accessibility of organic compounds) software package (https://www.molecular-networks.com/products/sylvia).

Using MOLGEN, we were able to generate libraries of α-amino acids (of the sort used by biology in constructing proteins, with diverse side chains on the α-carbon, and as opposed to β-, γ- or δ- *etc.* amino acids) [8] and nucleoside analogues (for our purposes, molecules enabling the attachment of a functional group such as a nucleobase which could provide non-covalent recognition motifs (though these could, in principle, be extremely heterogeneous, allowing recognition by many manners of intermolecular interaction), and also provide at least two stable functional groups that would still be stable when involved in covalent linkage in a polymer) [9]. For the second step, analysing the obtained libraries, we used molecular descriptors representing properties that might be involved in selection processes during chemical and early biological evolution.

Specifically, for the amino acids we explored and tested a model for biology's selection of the coded amino acids proposed by Philip & Freeland [50], based on their hydrophobicity, size and charge, represented by the partition coefficient (log*P*), van der Waals volume (*V*_vdw_) and acid dissociation constant (p*K*_a_). This method, also called adaptive analysis, gives insight to the adaptive properties of the amino acid alphabet. After generating and culling a very large set of possible α-amino acids and calculating their three key chemical properties, randomly sampled sets of α-amino acids were compared with the standard biologically coded amino acid alphabet with respect to range and evenness to the above-mentioned three properties (log*P*, *V*_vdw_ and p*K*_a_) [15].

For the nucleoside analogues, our approach broke new ground because there was as yet no established theory as to how to generate a nucleoside analogue, other than trial and error based on structure extension. Thus, we conducted a very broad analysis allowing a wide variety of fuzzy formulae, including C, H, N and O, although S could be incorporated, in principle, by atom substitution for O, which is another feature of this approach. As much as computational power may be saved by representing a nucleobase ‘B’ in a generic nucleoside analogue structure by an arbitrary univalent atom, divalent O can be replaced by divalent S without undertaking a complete *de novo* structure generation process; however, to do this, symmetry groups need to be taken into account.

## 3. Applications

We review here briefly the results of our previous research on amino acids and nucleoside analogues.

### (a) Amino acids

α-Amino acids are fundamental to biochemistry as the monomeric building blocks with which cells construct proteins according to genetic instructions. However, the 20 amino acids of the standard genetic code represent a small fraction of the number of α-amino acid chemical structures that could plausibly play such a role, both from the perspective of the natural processes by which life emerged and evolved and from the perspective of human-engineered genetically coded proteins. Efforts to describe the structures composing this broader set, or even estimate its size, had previously been hampered by the complex combinatorial properties of organic molecules. Estimates of the number and types of coded amino acids which could have been available to primitive biological systems are almost universally smaller than the actual coded set [51]. The principal reasons for this discrepancy are that abiological mechanisms for the exploration of structure space are inefficient (because abiological mechanisms are limited by reagents and mechanisms) and biological mechanisms are directed in ways we cannot efficiently model because they are history-dependent (e.g. because biology may discover new pathways which, however inefficient initially, may become obvious biotransformations after sufficient natural selection).

**Figure 1.**
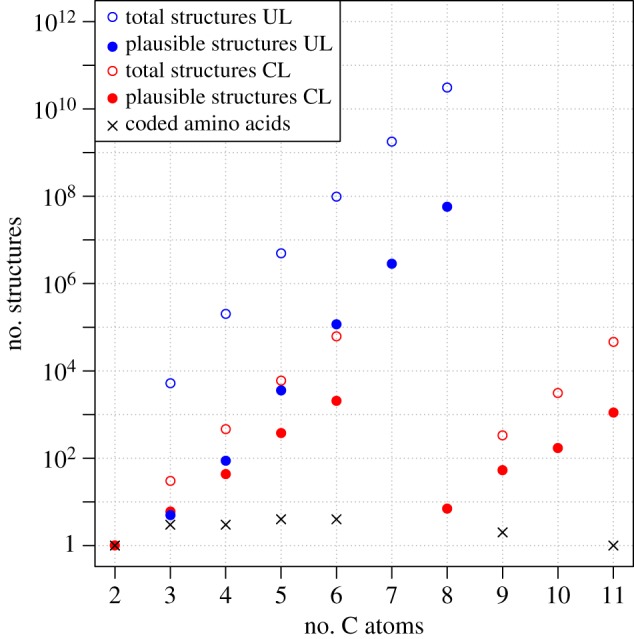
Sizes of the UL and CL α-amino acid libraries calculated during our previous studies. In order to reduce the total set of mathematically possible structures to structures which are chemically plausible, a list of 156 forbidden substructures was compiled.

In exploring amino acid space, we generated two virtual amino acid libraries using two different approaches. The first was a larger ‘unique’ library (UL) of 121 044 structures limited at an upper bound of six carbon atoms, which covered the space of molecular formulae as completely as possible, and the second was a smaller ‘combined’ library (CL) of 3846 structures, which included all coded amino acids containing up to 11 carbon atoms (i.e. tryptophan). The CL approach was developed as it was clear a UL approach would give an unwieldy structure set of the order of 10^12^ α-amino acid isomers up to, and including, 11 carbon atoms so as to include tryptophan. It should be borne in mind that these are structural isomer counts, and that the number of stereoisomers represented by these libraries is significantly higher, typically by a factor of 2–10 over this molecular weight range. Figure 1 shows the composition of these libraries itemized by the number of carbon atoms. A detailed description of the library design methods and the badlists used to derive these libraries has been published [8].

**Figure 2.**
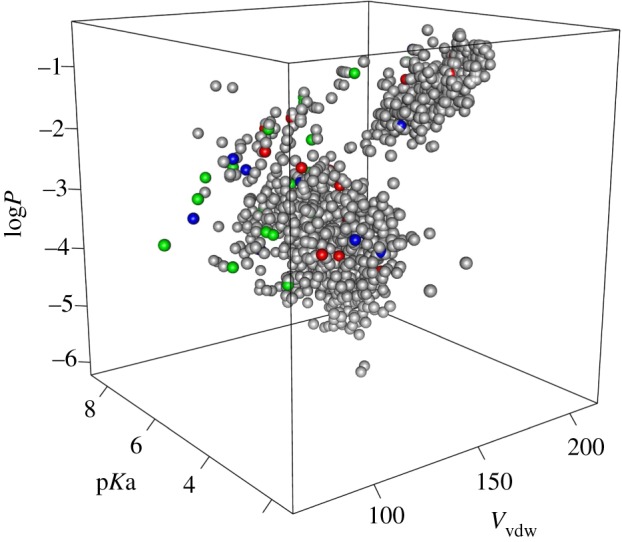
Chemical space of computed α-amino acids, represented by size, hydrophobicity and charge in terms of van der Waals volume (*V*_vdw_), partition coefficient (log*P*) and side chain acid dissociation constant (p*K*_a_). Green spheres represent the 20 coded amino acids, blue and red spheres show two of the rare ‘better’ sets. The cluster at top-right is formed by somewhat larger and more hydrophobic aromatic compounds.

We later [15] used the CL approach from Meringer *et al.* [8] to verify a conjecture on the optimality of the encoded amino acid alphabet formulated by Philip & Freeland [50]. These authors hypothesized that size, hydrophobicity and charge are the three physico-chemical properties of amino acids most likely to be responsible for their selection during the evolution of the genetic code. Figure 2 shows a mapping of our virtual amino acid library into a three-dimensional space defined by these properties. An adaptive set of amino acids was defined as one whose members thoroughly cover these physico-chemical properties. Using this metric, we compared the encoded amino acid alphabet with random sets of amino acids sampled from our virtual library. We further computed the heats of formation of all isomers to attempt to estimate the biosynthetic cost to a hypothetical organism using an alternative set. Sets that cover this chemistry space better than the genetically encoded alphabet were extremely rare and always energetically more costly. Further analysis of the computed more-adaptive sets revealed common features and anomalies. These computations were interpreted as evidence that the set of 20 amino acids found within the standard genetic code is the result of considerable natural selection. The amino acids used for constructing coded proteins may represent a largely global optimum, such that any aqueous biochemistry would use a very similar set [15].

### (b) Nucleotide analogues

**Figure 3.**
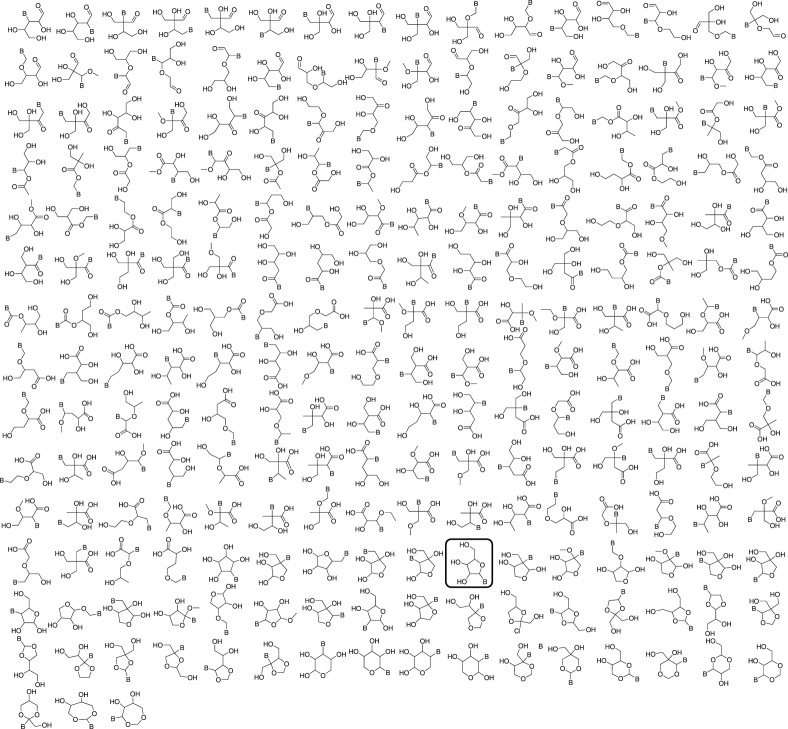
Enumerated set of nucleoside-like isomers of the natural ribosides. The structure corresponding to the natural ribosides is highlighted in black. (Reproduced with permission from [9].)

Ribonucleic acid (RNA) is one of the two nucleic acids used by extant biochemistry and plays a central role as the intermediary carrier of genetic information in transcription and translation. These roles, together with various catalytic and sensing functions of RNA in cellular processes (e.g. ribozymes and riboswitches), have been suggested to be functional remnants from a previous ‘RNA world’ biochemical state. If RNA was involved in the origins of life, it should have a facile prebiotic synthesis. A wide variety of such syntheses have been explored [52–55]. However, to date no one-pot reaction has been shown capable of yielding RNA monomers from likely prebiotically abundant starting materials, though this does not rule out the possibility that simpler, more easily prebiotically accessible nucleic acids may have preceded RNA. Given structural constraints, such as the ability to form complementary base pairs and a linear covalent polymer, a variety of structural isomers of RNA could potentially function as genetic platforms [56]. To give some sense of the size of this ‘alternative nucleic acid’ structure space, all the potential structural isomers of the ribosides (BC_5_H_9_O_4_, where B is nucleobase) that could potentially serve as monomeric building blocks of nucleic acid-like molecules were thus enumerated using the structure generation software (figure 3) [9].

Molecules were selected based on their likely stability under biochemically relevant conditions (e.g. moderate pH and temperature) and the presence of at least two functional groups, allowing the monomers to be incorporated into linear polymers. The resulting 227 structures were then evaluated using molecular descriptors derived from QSPR studies and predicted physico-chemical properties. Several databases were queried to determine whether any of the computed isomers had been synthesized previously, which showed that very few of these computed isomers had been described. Again, this is the structural isomer count, the number of stereoisomers was approximately four times larger, although the set includes a number of isomers that are achiral and/or prochiral. Based on these results, two broad conclusions could be drawn. First, ribonucleosides may have competed with a multitude of alternative structures whose potential proto-biochemical roles and abiotic syntheses remain to be explored. Second, based on QSPR analysis, the natural ribosides are among the most volumetrically compact isomers, and this may have been a factor contributing to their selection by biology.

**Figure 4.**
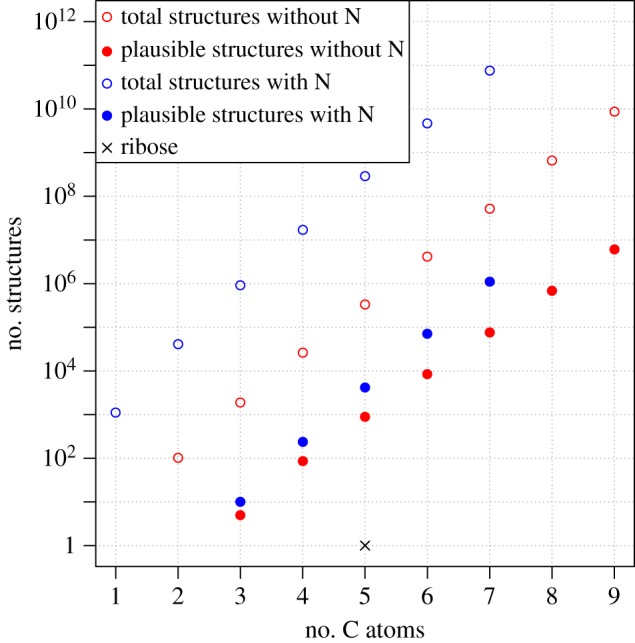
Numbers of nucleoside analogue structures as a function of the number of carbon atoms in the molecule. Enumeration is based on the formula spaces C_*n*_H_5−(2*n*+1)_O_2–4_B for structures lacking nitrogen and C_*n*_H_5−(2*n*+3)_N_1–2_O_0–4_B for those structures including nitrogen.

The formula representing these 227 riboside isomers is of course only one out of many which could conceivably be used to construct a nucleoside analogue. A variety of compounds with novel compositions are known in the literature which have good base-pairing properties (e.g. [57–59], in addition to the deoxynucleosides of DNA). We have since computed the structural space of nucleosides beyond C_5_H_9_O_4_B, which is as expected much larger (figure 4).

As was observed in the amino acid libraries, there is a roughly predictable exponential growth in the number of structures with increasing number of carbon atoms, and a considerable discrepancy (by a factor of 10^3^–10^5^) in the corresponding numbers of mathematically possible and chemically plausible structures. Further analysis of these results is ongoing.

## 4. Conclusion and outlook

It is clear that organic chemical structure space is very large, and that abiotic, and possibly prebiotic, chemistry sampled a significant but relatively small subspace of this set. Terrestrial biology also samples this large space, but again in a relatively limited fashion.

As this space is so large, and its coverage by abiotic and biological chemistry so poorly explored, structure generation offers a relatively cohesive and facile way to explore molecular possibilities *in silico*, which may be further used to direct real-world synthesis, observation and analysis. The types of questions enabled by these methods are of fundamental interest to the natural and physical sciences, and in particular allow for exploration of how life originated and might be instantiated beyond the Earth and in the laboratory.

According to our analyses so far, the set of coded amino acids and ribonucleotides used by biology do indeed appear to be highly non-random and by some metrics highly optimal, suggesting a significant amount of natural selection over the course of biochemical evolution. If this is the case, then chemists attempting to understand the chemical origins of life may do well to explore areas of organic chemical space which are not populated by the compounds used in modern biochemistry.

We are planning to extend and refine our studies of the chemical space of amino acids and nucleotide analogues. Possible directions would be to enlarge our amino acid libraries, e.g. by taking the so-called 21st and 22nd proteinogenic amino acids, selenocysteine and pyrrololysine, into account for library design, or even more amino acid structures that result from post-translational modifications. The adaptive analysis can be extended to cover sets of more or less than 20 members. Ongoing analysis of the structural space of nucleosides beyond C_5_H_9_O_4_B has already been mentioned above.

There are still more compound classes to be explored, for example lipids, which may hold interesting surprises with respect to the potential of other compounds to carry out the functions required by cell membranes. Another example is the chemical space underlying the intermediates of the reverse tricarboxylic acid cycle. A corresponding library has recently been generated [60] and is about to be analysed. Furthermore, the use of chemical reaction iteration approaches to understand not just the types of compounds which are structurally possible, but which are most easily accessed by abiotic and biological chemistry will undoubtedly greatly refine our conception of life's choice of chemical components.

## Data accessibility

This article has no additional data.
